# Real-World, National Study of Palbociclib in HR+/HER2− Metastatic Breast Cancer: A 2.5-Year Follow-Up PALBO01/2021

**DOI:** 10.3390/diagnostics15091173

**Published:** 2025-05-05

**Authors:** Cristina Marinela Oprean, Larisa Maria Badau, Ramona Petrita, Mircea Dragos Median, Alis Dema

**Affiliations:** 1 ANAPATMOL Research Center, ‘Victor Babes’ University of Medicine and Pharmacy of Timisoara, 300041 Timisoara, Romania; cristina.oprean@umft.ro (C.M.O.); dema.alis@umft.ro (A.D.); 2 Department of Oncology, ONCOHELP Hospital Timisoara, 300239 Timisoara, Romania; 3 Department of Oncology, ONCOMED Outpatient Unit Timisoara, 300239 Timisoara, Romania; 4 Hygiene Discipline, “Victor Babes” University of Medicine and Pharmacy, 300041 Timisoara, Romania; 5 Biometrics Unit MDX Research SRL Vasile Voichita 1-3 SAD2, Building C, 300633 Timisoara, Romania; ramona.petrita@mdxresearch.eu; 6 Gynecologic Oncology Department, Filantropia Clinical Hospital Bucharest, 011171 Bucharest, Romania; dragos.median@gmail.com

**Keywords:** breast cancer, cyclin-dependent kinase 4/6 inhibitor, palbociclib, real-world, Her2 low, Ki67

## Abstract

**Background:** Palbociclib, when combined with endocrine therapy, represents a valuable treatment option for patients diagnosed with hormone receptor (HR) positive/human epidermal growth factor receptor 2 (HER2) negative advanced breast cancer (BC) or metastatic breast cancer (MBC). Approved in Europe following phase II/III trials, it became the first CDK4/6 inhibitor used alongside hormone therapy. Available real-world data demonstrate the strong performance of Palbociclib in unselected, heavily pretreated patient groups. Our retrospective, observational, multicenter study, conducted in six Romanian institutions during a follow-up period of 2.5 years, aimed to assess Palbociclib’s safety and effectiveness in clinical practice. **Objectives:** The primary endpoints included response rate such as overall response rate (ORR), duration of response (DOR), disease control rate (DCR) and best clinical response (BCR), progression free survival (PFS) and overall survival (OS). The secondary objectives focused on treatment duration with aromatase inhibitors (AI) or fulvestrant and subsequent therapies after disease progression. Grade 3/4 adverse events were individually recorded. Exploratory analysis evaluated the potential predictive biomarkers such as Ki67, lower levels of HER2 expression (HER2-low), and histological or luminal subtype. **Methods:** Approximately 650 patients were planned for inclusion. PFS and OS were analyzed via the Kaplan–Meier method, with median times, 1- and 2-year estimates, and 95% confidence intervals reported. **Conclusions:** This study supports the integration of clinical trial evidence into real-world settings, enhancing patient selection and treatment personalization.

## 1. Introduction

**Figure 1:**
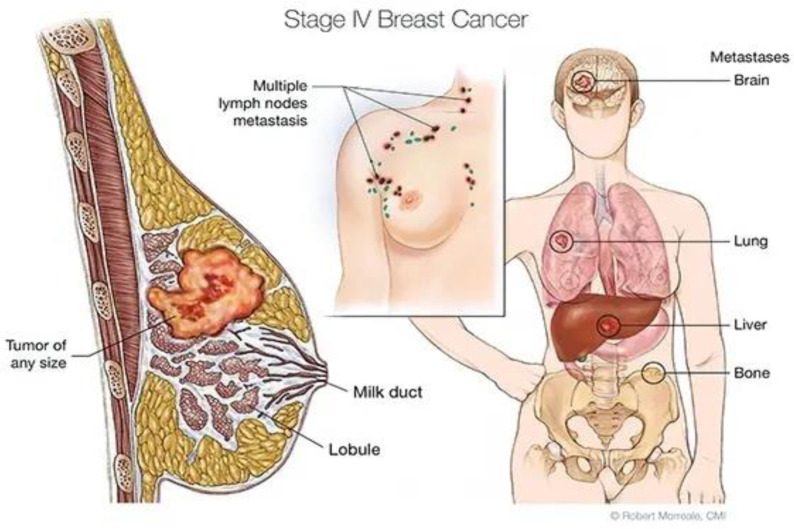
BC typically begins in the ducts or lobules. In MBC, it has disseminated to distant organs such as the bones, lungs, liver, or brain.

Metastatic breast cancer (MBC) is the most advanced stage of breast cancer and is where the disease has spread to distant sites beyond the axillary lymph nodes (Figure 1) [1]. At the European level, MBC occurs in up to 20–30 percent of subjects diagnosed with breast cancer [2]. Currently, the median overall survival (OS) for patients with MBC is approximately 2 to 3 years in developed countries, but lower in developing countries [2,3]. In Romania, 8900 new cases of breast cancer (BC) are diagnosed every year, with 80% being diagnosed in an advanced stage of the disease (II, III, IV). Furthermore, after initial breast cancer treatment, approximately 50% will develop MBC [4].

Treatment for MBC focuses on prolonging survival and elevating quality of life through specific approaches tailored to the pathological characteristics of the cancer, hormone receptor (HR), human epidermal growth factor receptor 2 (HER2) status, and other factors. Worldwide, the BC HR-positive/HER2-negative is the foremost common category with an age-adjusted rate of 87.2 new cases per 100,000 women, based on 2016–2020 cases [5]. The metastatic breast cancer cells are characterized by phenotypic differences, and different genetic mutations leading to treatment resistance [6].

Currently, CDK 4/6 inhibitors (Palbociclib, Ribociclib and Abemaciclib) represent the standard therapeutic approach for advanced HR-positive and HER2-negative BC. As of 9 November 2016, IBRANCE^®^ (Palbociclib) was the first CDK 4/6 inhibitor approved in Europe for use alongside letrozole in first-line treatment or with fulvestrant for women previously treated with endocrine therapy, supported by data from the PALOMA-1, PALOMA-2, and PALOMA-3 studies [6,7].

CDK4/6 inhibitors have shown effectiveness in both first-line [8,9,10] and endocrine-resistant settings, as demonstrated by numerous phase III randomized studies [11]. Even though randomized trials offer the strongest evidence, integrating their results into clinical practice can be challenging.

Palbociclib, when combined with endocrine therapy, represents a valuable treatment option for patients diagnosed with HR-positive/HER2-negative advanced BC or MBC. It remains a major challenge to identify the patients who are likely to benefit the most from Palbociclib therapy. Available real-world evidence demonstrates the strong performance of Palbociclib in unselected, heavily pretreated patient groups.

The shift from evidence gathered in randomized clinical trials to everyday clinical practice began with real-world studies designed to inform prescribing decisions. The clinical effectiveness and treatment approaches of Palbociclib have been evaluated in real-world clinical settings in Italy, Germany, France, the UK, Spain, the USA, Canada, Argentina and China [12,13,14,15,16].

By performing an observational study in a real-world clinical setting, based on medical charts, valuable data can be obtained on both the safety and efficacy of Palbociclib.

Starting with August 2018, treatment with IBRANCE was reimbursed in Romania, according to the amended list of therapeutic protocols issued by the Ministry of Health and National Health Insurance System.

## 2. Materials and Methods

### 2.1. Trial Design Overview

This study uses an observational cohort design. The design of this study captured data on patients who retrospectively initiated therapy with Palbociclib in multiple Institutions across Romania. Prescription data collection started upon notification of the date of market launch in Romania (August 2018) and continued for approximately 2.5 years, or until the target sample size was achieved (whichever was the soonest). This clinical study was conducted in six investigational centers located in Romania. The final cohort size is estimated to be around 650 patients. The site personnel captured all data electronically at the study site via electronic case report forms (e-CRFs). Data from external sources (such as laboratory data) were entered into electronic data capture (EDC) system or imported and combined into the final analysis datasets.

Palbociclib was regarded as first-line treatment (LOT1) when given to patients with no previous systemic therapy for MBC or if at least one year had passed since the completion of adjuvant endocrine therapy. If given after disease progression on first-line treatment for MBC or following relapse at or within one year of finishing adjuvant hormone therapy, Palbociclib was considered second-line therapy (LOT2).

Clinical- and disease-related characteristics, including factors like age, menopausal status, stage at initial diagnosis, pathological subtype, tumour grade, molecular subtype, HR, HER2, Ki67 value, metastatic disease status (de novo or recurrent), type of metastatic site and number of organ metastasis information was collected. History of previous treatments for BC such as adjuvant or neoadjuvant chemotherapy, hormonotherapy, radiotherapy and also disease-free interval for recurrent MBC was collected.

A synopsis of prior and baseline comorbidities and concomitant medication information was also collected. Prior, concomitant, and follow-up medication use was outlined descriptively based on the number and percentage of participants taking each medication across different therapeutic classes. Multiple use of the same medication by a participant was counted only once. All concomitant medications were noted by the investigator during the study.

The safety-evaluable population included the Intention-to-Treat (ITT) cohort, comprising all participants who received at least three treatment cycles (each lasting 21 days) of Palbociclib.

The efficacy analysis was carried out in Per-Protocol (PP) population, which included only participants who did not record any major protocol deviations.

The follow-up period was defined as the duration from the beginning of a treatment regimen until the first occurrence of disease progression, death, or the last recorded medical documentation.

### 2.2. Eligibility Criteria

Patients meeting the eligibility criteria described in Table 1 were included.

**Figure 2:**
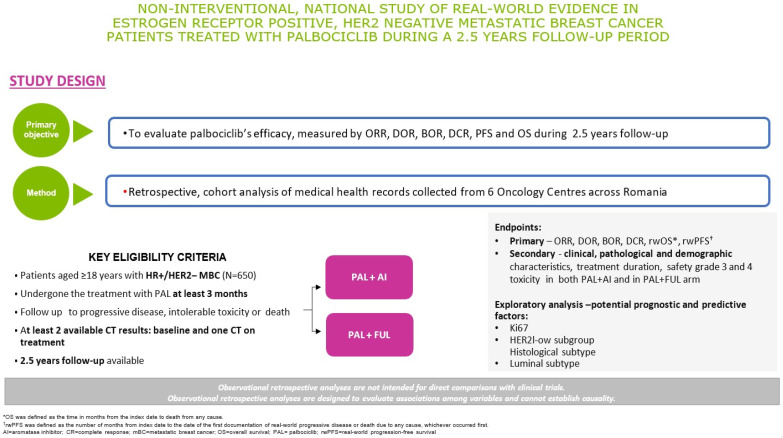
Trial design overview.

The trial design is summarized in Figure 2.

### 2.3. Sample Size. Procedure and Data Collection

#### 2.3.1. Sample Size

A number of 650 patients were planned to be included in the present study. The final cohort size and the duration of recruitment were influenced by the level of prescribing of Palbociclib by oncologists from the investigational centers. Each patient enrolled was identified by a three-digit code and patient initials which are the only identification elements and will be used only for the purposes of this study.

Clinical data were collected during thirteen visits as follows: date of diagnostic, followed by visits performed at 30 days, 60 days, 90 days, 120 days, 270 days, 360 days, 450 days, 540 days, 630 days, 720 days, 810 and 900 days.

Data quality and completeness were first assessed in relation to the data analysis. If any participant has missing data for one or more variables, even after the query has been addressed, the missing values were not substituted. In the event of a participant violating the inclusion/exclusion criteria, their data were not included in the analysis.

#### 2.3.2. Procedure and Data Collection

During November 2021–May 2023, the medical charts and medical databases were analyzed in order to search for potential eligible participants.

Demographic data, such as age, urban or rural environment, gender, race, ethnicity, medical history and disease history were collected. Clinical data such as menopausal status, stage at initial diagnosis, metastatic disease status (de novo or recurrent), type of metastatic site and number of organ metastasis information were collected and recorded. Pathological subtype, tumour grade, molecular subtype, HR, HER2, Ki67 value were also collected. History of previous treatments for BC such as adjuvant or neoadjuvant chemotherapy, hormonotherapy, radiotherapy and also disease-free interval for recurrent MBC was collected. A synopsis of prior and baseline comorbidities and concomitant medication information was also collected.

The follow-up period is defined as the duration from the beginning of a treatment regimen until the first occurrence of disease progression, death, or the last recorded medical documentation.

**Figure 3:**
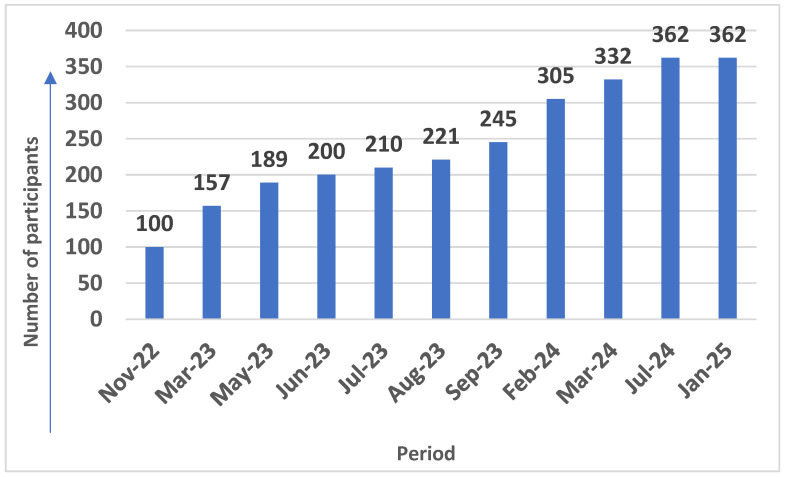
The patient data collection progress.

The patient data collection progress is summarized in Figure 3. We officially closed data collection on 17 January 2025.

### 2.4. Objectives and Methods of Assessment

#### 2.4.1. Primary Objectives

Our study’s primary objectives were to determine pathological and clinical features of HR-positive/HER2-negative MBC that are associated with highest efficacy of Palbociclib, evaluated through response rates such as overall response rate (ORR), duration of response (DOR), disease control rate (DCR) and best clinical response (BCR), progression-free survival (PFS) and OS. The response rate was assessed, retrospectively, by computer tomography scan using the RECIST V1.1 criteria.

The efficacy analysis was carried out in PP population, which includes only participants who did not record any major protocol deviations.

#### 2.4.2. Secondary Objectives

The secondary objective is to determine the median duration of the treatment with AI in first-line and with fulvestrant in second-line therapy. Moreover, we are looking to characterize the type and number of the subsequent lines of treatments after progression disease under treatment study.

#### 2.4.3. Safety Objectives

Safety was assessed by the clinical symptoms from the medical charts and the laboratory parameters (Hematology and Biochemistry). We assessed grade 3 and 4 clinical toxicities (fatigue, upper respiratory infection, nausea) and biological toxicities (neutropenia, anemia, thrombocytopenia, elevated liver enzyme, alkaline phosphatase, bilirubin). Any other toxicities associated with treatment, graded 3 or 4, were documented.

The safety evaluable individuals included ITT cohort consisting of all the participants who received Palbociclib.

Safety assessment included the monitoring of adverse events of grade 3 and 4 severity and other toxicities associated with treatment, as defined in Common Terminology Criteria for Adverse Event (CTCAE) v5.0. The safety variables included also all laboratory assessments performed for each participant throughout the follow-up period.

All investigators involved in this study were responsible for reporting all adverse events that occurred during the study. The minimum required information was:

- Date of onset;
- The degree of the event’s severity (Mild/Moderate/Severe);
- If the adverse event is serious (SAE);
- The causality assessment with the drug;
- Any other medical interventions performed by Investigator (e.g., changing concomitant medications);
- Status of the event at the moment of the report (Ongoing, Resolved);
- If resolved, stop date of the event.

#### 2.4.4. Exploratory Variables

A subset of patients will be included in the exploratory analysis to determine whether other biomarkers, such as Ki67, lower levels of HER2 expression (HER2-low), histological or luminal subtype, are prognostic and/or predictive of response to Palbociclib in first or second line of therapy.

#### 2.4.5. Measurement Methods and Outcomes

The primary endpoints are:

- ORR is defined as the percentage of patients achieving a confirmed complete response (CR) or partial response (PR);
- DOR is defined as the period of time between the initial documentation of tumour response and the subsequent confirmation of disease progression (PD);
- DCR is defined as the percentage of patients who achieve the best overall response, (CR), (PR), or stable disease (SD), that is maintained for at least 12 weeks;
- BCR is defined as the best imaging response during the treatment time;
- Clinical Benefit Rate (CBR) is defined as the proportion of patients with no disease progression after 6 months of therapy;
- PFS is defined as the time from initiation of treatment to occurrence of PD or death;
- OS is defined as the duration from the start of treatment until death from any cause. For patients who are still alive, follow-up will be censored at the last recorded contact date or the most recent date they were known to be alive. The OS follow-up was conducted at 12, 24, and 30 months (2.5 years). If data were available beyond the 2.5 years, OS was collected also at 33 months from the initiation of Palbociclib.

All these objectives were evaluated by RECIST V1.1 criteria, using CT scan results collected retrospectively at multiple time points during follow-up period, respectively, every three months. The follow-up period was 2.5 years.

#### 2.4.6. Laboratory Variables

The data were, retrospectively, collected from each patient’s medical chart (Table 2).

#### 2.4.7. Demographics and Baseline Characteristics

Subject demography consists of age at screening, race, ethnicity and gender.

Date of first diagnosis and date of first treatment were documented as records of disease history in the EDC system.

Tumour grading was performed based on WHO criteria, which consider the proportion of tubular structures versus solid areas, mucin production, nuclear abnormalities, and mitotic rate [16,17].

The Tumour–Nodules–Metastasis (TNM) classification, applied clinically and/or pathologically, was used to determine the disease stage. The molecular subtype was categorized based on the 2017 St. Gallen Consensus, considering ER, PR, Ki67, and HER2 status [18]. These parameters were derived from the results of immunohistochemistry examinations. ER and/or PR were regarded as positive when the value exceeded > 1%. A cut-off value of 20% for Ki67 was used to differentiate between luminal A and luminal B tumours. To differentiate between HER2-positive and HER2-negative tumours, immunohistochemistry and in situ hybridization test results were collected for each patient.

Patients with bilateral breast cancer were included in a separate subgroup. The immunohistochemistry examination results and the histopathological subtype was collected for both tumour foci, if available.

For documenting medical history, any previous or concomitant diseases present before screening were entered into the EDC.

### 2.5. Study Drug

The initial dose was 125 mg of Palbociclib taken once daily for 21 consecutive days, followed by 7 days off the treatment to complete a 28-day cycle (Schedule 3/1). The following two dose reduction levels were defined: Palbociclib 100 mg once daily or 75 mg once daily, administered orally for 21 consecutive days followed by 7 days off treatment.

Palbociclib was administered in combination with an aromatase inhibitor or with fulvestrant in women who have received prior endocrine therapy.

Eligible patients are required to receive treatment for a minimum of three months.

### 2.6. Statistical Methods

An approximate number of 650 patients was planned to be included in the present study. The advantage of a larger sample size is the fact that it gives more reliable results with greater precision and power.

For quantitative variables (e.g., demographic data), if they are normally distributed, they were presented as mean ± standard deviation (SD); otherwise, the median, minimum, maximum, and interquartile range were reported. Qualitative variables were evaluated using frequencies and percentages.

Time-to-event outcomes (PFS, OS) were analyzed using the Kaplan–Meier method. The median time to event as well as the 1- and 2-year estimates was reported along with 95% confidence intervals (CI). CI will be calculated using the Clopper–Pearson method.

The quality and completeness of the collected data will be preliminarily assessed in comparison to the data analysis. If a participant was missing information for one or more variables, even after the query has been resolved, the missing data will not be imputed. If a participant is found to have violated the inclusion/exclusion criteria, the corresponding data will be excluded from the analysis.

The number and percentage of participants that completed at least 3 treatment cycles (each with 21 days administration) will be presented for each group: LOT1 and LOT2.

Reasons for discontinuation from the treatment period as recorded on the eCRF were summarized (number and percentage) by treatment group for all participants. Participants excluded from the safety and efficacy (e.g., Full Analysis Set, Per-Protocol Set) populations or other analysis populations were listed.

The Investigator will not implement any deviation from the Clinical Study Protocol without agreement from the Sponsor, except where necessary to eliminate an immediate hazard to clinical investigation participants.

Demographic and other baseline characteristics, including gender, age, race, ethnicity, medical history, disease history will be summarized by treatment lot for the safety set using descriptive statistics. Descriptive summaries included frequency tables for all categorical response variables and number, mean, SD, minimum, and maximum for all continuous variables.

Prior, concomitant, and follow-up medication use were summarized descriptively by the number and percentage of participants receiving each medication within each therapeutic class.

The response rate was assessed by CT scan and evaluated retrospectively in order to evaluate best response rate and duration of response.

## 3. Discussion

Real-world data play a vital role in providing evidence on the safety and efficacy of drugs in everyday clinical settings, especially for patients who fall outside the inclusion or exclusion criteria of clinical trials.

The provided information indicates that the current study presents a favorable balance between risks and benefits. The overall efficacy and safety of Palbociclib have been thoroughly documented in the literature and supported by various clinical studies [9,19].

Palbociclib significantly improved PFS and CBR when combined with letrozole as initial endocrine-based therapy in postmenopausal women. Additionally, in randomized clinical trials, it extended PFS and OS when added to fulvestrant in women who had progressed on prior endocrine therapy. The tolerability profile was manageable, with neutropenia being the most common occurrence, yet without a negative impact on quality of life.

Real-world data confirm the good performance of Palbociclib in unselected, heavily pretreated patients. When combined with endocrine therapy, Palbociclib represents a valuable emerging option for patients with HR-positive/HER2-negative advanced BC or MBC.

Currently, Palbociclib is approved in more than 95 countries and has been prescribed to nearly 340,000 patients globally [20]. Pre-marketing data will usually provide little information on drug utilization and safety post-marketing. Therefore, additional data are needed to further assess the drug’s safety and efficacy profile, as well as to gain a broader understanding of practice patterns and Palbociclib outcomes. This could help determine the optimal treatment sequence to maximize patient benefit while minimizing toxicity.

The overall safety profile of Palbociclib is based on pooled data from 872 patients who received the drug in combination with endocrine therapy (N = 527 in combination with letrozole and N = 345 in combination with fulvestrant) in randomized clinical studies in HR-positive, HER2-negative advanced BC or MBC [21].

Real-world evidence studies demonstrate that Palbociclib, when administered in routine clinical practice as a first-line or later-line treatment, yields efficacy and toxicity outcomes consistent with those observed in pivotal trials. [20,22].

These real-world findings may help clinicians tailor by luminal subtype and the value of Ki67. In a recent study conducted by Shao, patients with Ki67 levels of ≥14% experienced a marginally shorter PFS compared to those with Ki67 levels below 14% (*p* = 0.062) [23]. Patients with Ki67 levels of 30% or greater experienced significantly shorter PFS compared to those with Ki67 levels below 30% (*p* = 0.048), while PR levels of 20% or higher were associated with prolonged PFS. Furthermore, changes in Ki67 or PR levels from primary tissue to metastatic lesions were associated with PFS. Patients with Ki67 levels of ≥14% or ≥30% experienced shorter PFS compared to those with levels below these thresholds (*p* = 0.024 and *p* < 0.001, respectively). Additionally, changes in Ki67 or PR levels from primary to metastatic lesions were associated with progression-free survival (PFS). When considering both markers simultaneously, significant differences were observed between the cohorts. Patients with Ki67 < 14% and PR ≥ 20% demonstrated considerably longer PFS compared to those with Ki67 ≥ 14% and PR < 20%. Moreover, patients with Ki67 < 30% and PR ≥ 20% experienced significantly longer PFS than those with Ki67 ≥ 30% and PR < 20%. Also, in the AI cohort, patients with Ki67 < 14% and PR ≥ 20% had significantly longer PFS compared to those with Ki67 ≥ 14% and PR < 20%. Similarly, women with Ki67 < 30% and PR ≥ 20% demonstrated significantly longer PFS than those with Ki67 ≥ 30% and PR < 20% [24].

The IRIS study (Ibrance Real World Insights) is a multinational study designed to evaluate Palbociclib in patients with HR-positive/HER2-negative advanced BC or MBC in a real-world setting across several countries globally. Clinical outcomes in Argentina indicate that the combination of Palbociclib with either letrozole or fulvestrant demonstrates favorable effectiveness, as reflected in improved PFS and OS rates. Across five European countries (Italy, Germany, the UK, Belgium and Switzerland) the median duration of follow-up from Palbociclib onset was 10.0 months for Palbociclib and AI, respectively, 7.2 months for Palbociclib and Fulvestrant patients. Reported progression-free rates for Palbociclib and AI at 12 months were 88.2% and 62.6% at 24 months. At 12 months, the OS rate was 97.7%, declining to 93.2% at 24 months. For patients receiving the combination of Palbociclib and Fulvestrant, the PFS rate was 81.1% at 12 months and 55.2% at 24 months, while the OS rates were 96.8% at 12 months and 85.2% at 24 months [12].

Additionally, when considering the interaction between pathological characteristics and the treatment line, the luminal B subtype was associated with a significantly poorer outcome (*p* = 0.043). In patients receiving endocrine therapy in combination with CDK4/6 inhibitors, PFS was inversely related to Ki67 levels, but not to PR expression. This finding suggests that tumour proliferation has a more pronounced impact on the effectiveness of the combined therapy than PR levels [12]. Since testing for Ki67 and PR is readily accessible, cost-effective, and practical, their role as predictors of PFS in patients treated with Palbociclib could be broadly applicable, warranting further validation of their prognostic value.

This study has certain limitations, primarily due to its retrospective design, which impacts the reliability of real-world data. Furthermore, in developing countries such as Romania, there is a possibility of underreporting adverse events. Additionally, it should be noted that applying findings from clinical trials to routine clinical practice can be challenging, as the strict, closely monitored conditions of trials, conducted within specific time frames, are often difficult to replicate in everyday medical settings.

Overall, this is the first real-world data on the use of Palbociclib in the Romanian population, showing effectiveness comparable to previously published real-world evidence. It has become the standard of care for first- and second-line treatment of HR-positive/HER2-negative advanced BC or MBC.

The results will be analyzed and interpreted in the context of existing studies and are expected to support the hypothesis that Palbociclib demonstrates consistent efficacy and safety across both clinical trials and real-world settings. Moreover, this study offers valuable insights into treatment patterns and patient outcomes within a national, multicenter framework, with particular relevance to Central and Eastern Europe, where real-world evidence remains limited.

## 4. Conclusions

This study aims to evaluate the response rate, clinical benefit, overall survival, progression-free survival, duration of response, and tolerability of Palbociclib in a real-world, non-trial setting over a 2.5-year follow-up period.

We anticipate that the long-term treatment outcomes observed across various lines of Palbociclib therapy will align with existing real-world evidence, supporting its sustained effectiveness and safety outside of clinical trials. These findings are expected to contribute valuable insights into patient selection and treatment optimization in everyday clinical practice.

Future studies should also investigate the molecular mechanisms of resistance and identify predictive biomarkers that could guide individualized treatment decisions. Prospective registries and longer follow-up periods will be essential to validate these findings and improve long-term patient management.

## Figures and Tables

**Table 1:** Eligibility criteria.

Inclusion Criteria
Adult women and men (≥ 18 years of age) with proven initial diagnosis of BC with evidence of loco-regional recurrent or metastatic disease not amenable to resection or radiation therapy. Documentation of histologically or cytologically confirmed diagnosis of BC with IHC of ER expression > 1% and/or PR expression >1% based on local laboratory results. Scoring of 0 or 1+ for HER2 protein expression by a validated immunohistochemistry assay or +1/+2 with negative HER2 amplification FISH/ISH ratio lower than 1.8 or HER2 gene copy less than 4.0. Eligible participants must have undergone treatment with Palbociclib for at least 3 months. Evaluable disease as defined per modified Response Evaluation Criteria in Solid Tumours (RECIST) V1.1 criterion (at least 2 entries). Premenopausal or postmenopausal status. 6.1. Patients who are not postmenopausal must have undergone treatment with LHRH agonist. 6.2. Postmenopausal status is defined as: (a) prior bilateral surgical oophorectomy; (b) spontaneous cessation of regular menses for at least 12 consecutive months; (c) in case of doubt serum estradiol <20 umol/l and follicle-stimulating hormone (FSH) levels >15 IU/L.
**Exclusion criteria**
Participants with advanced, symptomatic, visceral spread, such as patients with massive uncontrolled effusions (pleural, pericardial, peritoneal), (pulmonary lymphangitis, and over 50% liver involvement). Palbociclib treatment as part of a clinical trial or prescription prior to market approval (Nov 2016).

**Table 2:** Laboratory assessments.

Hematology	Clinical Chemistry	Liver Enzymes
Erythrocytes	Creatinine	Alkaline phosphatase
Mean corpuscular volume Hemoglobin Hematocrit	Glucose Urea	Aspartate aminotransferase
Neutrophils	Uric acid	Alanine aminotransferase
Eosinophils Basophils Lymphocytes	Total Bilirubin	Gamma-glutamyl transferase
Monocytes Platelets Leukocytes		

## Data Availability

Data are available upon request due to privacy and ethical restrictions.

## Abbreviations

The following abbreviations are used in this manuscript:

HR	Hormone receptor
HER2	Human epidermal grow factor receptor 2
BC	Breast cancer
MBC	Metastatic breast cancer
CDK 4/6	Cyclin-dependent kinase 4/6
OS	Overall survival
AI	Aromatase inhibitors
e-CRF	Electronic case report form
EDC	Electronic data capture
ITT	Intention-to-Treat
PP	Per-Protocol
ER	Estrogen receptor
PR	Progesterone receptor
RECIST	Response Evaluation Criteria in Solid Tumours
ORR	Overall response rate
DOR	Duration of response
DCR	Disease control rate
BCR	Best clinical response
PFS	Progression Free Survival
CTCAE	Common Terminology Criteria for Adverse Event
SAE	Serious adverse event
CR	Complete response
PR	Partial response
PD	Progression disease
SD	Stable disease
TNM	Tumour–Nodules–Metastasis
